# Treatment of Eczema

**Published:** 1884-10

**Authors:** 


### TREATMENT OF ECZEMA AND LEUCORRHŒA.

THE "Obstetric Gazette" states that in cases of eczema in which glyceroles and unguents have failed, the following formula has been successful:

[R] Chlorate of potassium, 30 grains,
Wine of opium, 50 grains,
Pure water, 1 quart.

This is applied by linen compresses covered with silk. In the event of much existing inflammation, this application should be preceded by warm sitz baths or cataplasms sprinkled with powdered carbonate of lime. In obstinate pruritis, associated with leucorrhœa, a teaspoonful of a mixture of equal parts of tincture of iodine and iodide of potassium, in a quart of warm tar water (tar holding the iodine in solution) used daily, night and morning, removes the pruritis and ameliorates the leucorrhœa. In fetid leucorrhœa two or three tablespoonfuls (in a quart of warm water morning and evening as an injection) of the following formula will be found useful:

[R] Chlorate of potassium, 13 parts,
Wine of opium, 10 parts,
Tar water, 300 parts,
White (or wine) vinegar 300 parts,
Tincture eucalyptus, 45 parts,
Salicylic acid, 1 part,
Salicylate of sodium, 20 parts.

One to 5 teaspoonfuls in a qrt. of warm water 3 times a day.

